# Insulin enhances metabolic capacities of cancer cells by dual regulation of glycolytic enzyme pyruvate kinase M2

**DOI:** 10.1186/1476-4598-12-72

**Published:** 2013-07-09

**Authors:** Mohd Askandar Iqbal, Farid Ahmad Siddiqui, Vibhor Gupta, Shilpi Chattopadhyay, Prakasam Gopinath, Bhupender Kumar, Siddharth Manvati, Noor Chaman, Rameshwar NK Bamezai

**Affiliations:** 1 National Centre of Applied Human Genetics, School of Life Sciences, Jawaharlal Nehru University, New Delhi 110067, India

**Keywords:** Insulin, Cancer, Metabolism, Lactate, Glycolysis, HepG2, PKM2

## Abstract

**Background:**

Insulin is tightly associated with cancer progression; however, mechanistic insights into such observations are poorly understood. Recent studies show that metabolic transformation is critical to cancer cell proliferation. Here, we attempt to understand the role of insulin in promotion of cancer metabolism. To this end, the role of insulin in regulating glycolytic enzyme pyruvate kinase M2 (PKM2) was examined.

**Results:**

We observed that insulin up-regulated PKM2 expression, through PI3K/mTOR mediated HIF1α induction, but significantly reduced PKM2 activity independent of this pathway. Drop in PKM2 activity was attributed to subunit dissociation leading to formation of low activity PKM2 oligomers, as assessed by density gradient centrifugation. However, tyrosine 105 phosphorylation of PKM2, known for inhibiting PKM2 activity, remained unaffected on insulin treatment. Interestingly, insulin-induced ROS was found responsible for PKM2 activity reduction. The observed changes in PKM2 status led to augmented cancer metabolism. Insulin-induced PKM2 up-regulation resulted in enhanced aerobic glycolysis as confirmed by PKM2 knockdown studies. Further, PKM2 activity reduction led to characteristic pooling of glycolytic intermediates and increased accumulation of NADPH; suggesting diversion of glucose flux towards macromolecular synthesis, necessary for cancer cell growth.

**Conclusion:**

The study identifies new PKM2-mediated effects of insulin on cancer metabolism, thus, advancing the understanding of insulin’s role in cancer.

## Background

Cancer cell metabolism differs from that of normal cells and is characterized by high glucose uptake and lactate production even in presence of sufficient oxygen, an observation made by Otto Warburg in 1920’s. The phenomenon has been referred to as *Warburg effect* or *aerobic glycolysis*[1] which forms the basis of ^18^fluorodeoxyglucose-positron emission tomography (FDG-PET) – a diagnostic technique used for clinical detection of cancer [2]. Although, inefficient in terms of ATP production, aerobic glycolysis, nonetheless, is vital to maintain macromolecule synthesis needed to produce daughter cells [3]. Further, aerobic glycolysis is believed to provide cancer cells a selective advantage to grow in regions with low oxygen concentration [4]. Considering the significance of aerobic glycolysis to cancer cells, metabolic behaviour is now perceived as a critical target for anti-cancer therapeutics [5,6]. Among the several glycolytic genes that are up-regulated in cancer [7], pyruvate kinase is of prime significance because of its key position in glycolytic sequence [8,9]. It catalyzes the last step of glycolysis i.e. conversion of phosphoenolpyruvate (PEP) to pyruvate with concomitant ATP production [10]. Out of four isoforms of pyruvate kinase (PK) in mammals- L, R, M1 and M2 [11]; tumor cells predominantly express M2 isoform [11]. Switch to this isoform is considered essential for aerobic glycolysis and tumor growth [12].

Activity of PKM2 is critical to determine a shift in metabolism required for tumor growth. Decrease in PKM2 activity causes pooling of glycolytic intermediates which are then shunted to pentose phosphate pathway (PPP) for NADPH generation for macromolecular synthesis, essential for cellular growth [8,9,11,13]. Therefore, decreased PKM2 activity is believed to favour rapidly proliferating cancer cells; however, the phenomenology of PKM2 activity regulation by external factors is not well understood. PKM2 exists in highly active (tetramer) and less active (dimer/monomer) oligomeric forms. The ratio of these forms decides the overall PKM2 activity in cellular milieu [11,14,15]. In cancer cells, disruption of active PKM2 tetramers results in accumulation of less active PKM2 monomers/dimers, thus, promoting anabolic synthesis [9,13]. Our previous work has shown how two missense mutations in PKM2 stabilized less active PKM2 form to promote cellular growth and polyploidy [16].

Evidences in recent years have suggested a crucial role of insulin in cancer cell growth and survival [17-20]. High levels of endogenous insulin among type-2 diabetics and non-diabetics have also been associated with increased cancer risk in epidemiological studies [21]; with highest risk for liver and pancreatic cancer [22-24]. Significantly, insulin receptor has been reported to provide resistance to IGF-1R targeted therapies [25]; emphasizing the importance of targeting insulin signalling. The role of insulin, however, in regulation of cancer cell metabolism is still obscure.

In this study, the role of insulin in promotion of cancer metabolism is examined. We show that insulin promotes cancer metabolism by upregulating PKM2 expression and decreasing its activity. Insulin-induced changes in PKM2 status directly resulted in amplification of cancer-metabolism-specific parameters like glucose uptake, lactate production, glycolytic pooling and macromolecular synthesis. Our results contribute to better understanding of the role of insulin in cancer metabolism and thus cancer progression.

## Results

### PKM2 is the predominant isoform of pyruvate kinase in cancer cells

**Figure 1:**
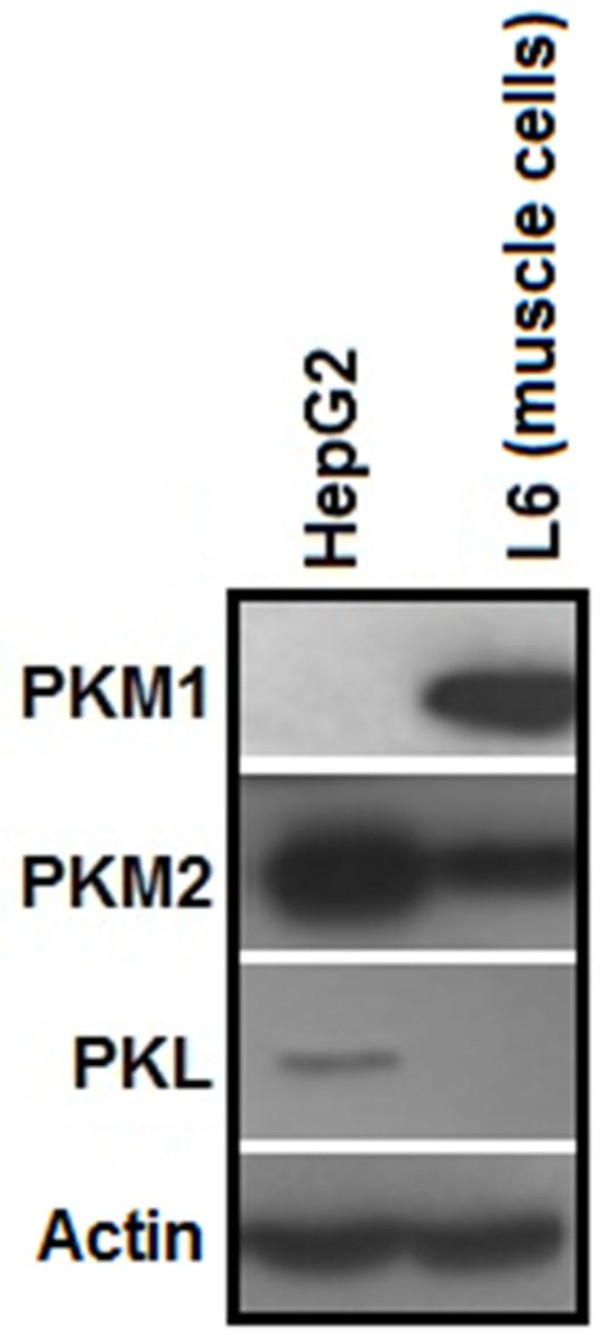
**PKM2 is the major isoform in HepG2 cells.** Immunoblot showing that PKM2 expression is many-fold higher than other PK isoforms in hepatoma cells. PKL, which is typically present in normal liver cells, is expressed at very low levels in cancerous HepG2 cell line. M1, which is present in energy requiring tissues such as brain and muscle, is completely absent in HepG2 cells. L6 cell line (rat skeletal muscle) is used as a positive control for M1 and a negative control for PKL isoform.

PK exists in four isoforms in mammals; therefore, isoform status of PK in HepG2, H1299 and PC3 cells was examined. Immunoblotting analysis in HepG2 showed that PKM2 was the predominant isoform in these cells with complete absence of PKM1 (Figure 1). Whereas, PKL isoform, which is predominant in normal liver cells, expressed at a much lower level in HepG2 cells (Figure 1). Similarly, PKM2 was the predominant isoform in H1299 and PC3 cells with negligible PKM1 expression (Additional file 1: Figure S1).

### Insulin upregulates PKM2 expression in a PI3K/mTOR dependent manner by inducing HIF1α expression

**Figure 2:**
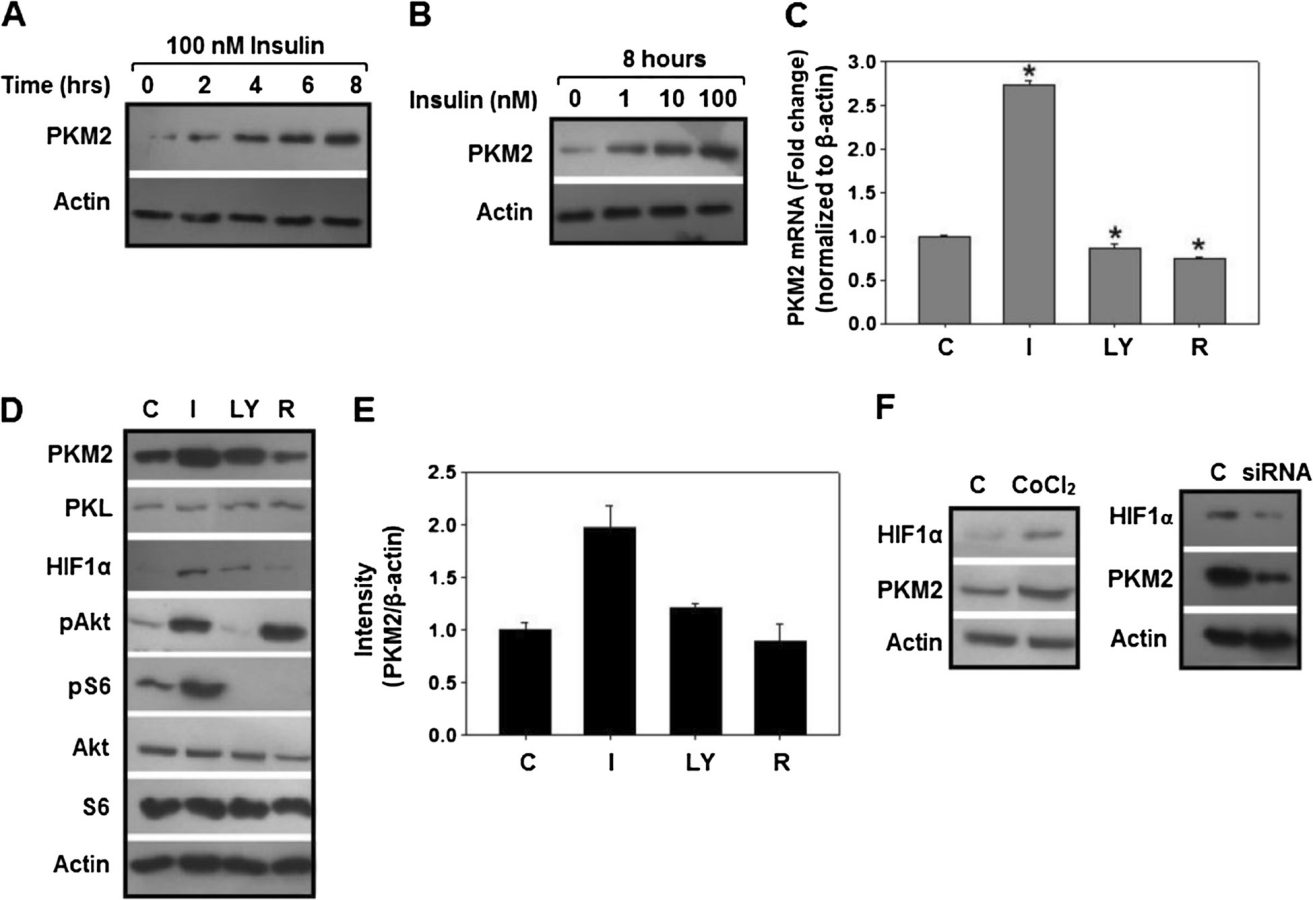
**Insulin increased PKM2 expression via HIF1α up-regulation, in a PI3K/mTOR dependent manner. (A)** Serum starved HepG2 cells were treated with 100 nM insulin for 0–8 hours, maximum increase in PKM2 protein observed after 8 hours of 100 nM treatment. **(B)** Dose dependent increase in PKM2 expression. **(C)** Total RNA and protein was extracted and subjected to real time PCR and immunoblotting respectively, PKM2 mRNA increased by ~2 fold in a PI3K/mTOR dependent manner (as LY294002 and rapamycin decreased PKM2 mRNA). For PI3K/mTOR inhibition, cells were pretreated with 50 μM LY294002 or 20 nM rapamycin for 30 minutes before stimulating cells with insulin. **(D)** Representative immunoblot showing insulin-induced up-regulation of PKM2 and its transcriptional regulator HIF1α in a PI3K/mTOR sensitive manner. However, PKL expression remained unchanged on insulin treatment. For PI3K/mTOR inhibition, cells were pretreated with 50 μM LY294002 or 20 nM rapamycin for 30 minutes. **(E)** Densitometric analysis was done using Alpha Imager EP densitometer (Alpha Innotech Corp., USA). **(F)** CoCl_2_ treatment (100 μM for 8 hours) resulted in accumulation of HIF1α with concomitant increase in PKM2 expression, siRNA mediated HIF1α silencing resulted in decreased PKM2 expression. These results suggest that insulin promoted PKM2 expression through HIF1α induction, in a PI3K/mTOR dependent manner. β actin was used as endogenous control in real time experiments and loading control in immunoblotting. mRNA data is expressed as mean ± SE. *P ≤ 0.05*.*

To examine the effect of insulin on PKM2 expression, serum starved HepG2 cells were treated with insulin for 0, 2, 4, 6 and 8 hours with maximum increase in PKM2 expression at 8 hours of insulin treatment (Figure 2A). Dose dependent effect of insulin on PKM2 expression revealed that 1 nM insulin (near physiological concentration) substantially induced PKM2 expression, however, maximum increase in PKM2 expression was observed with 100 nM insulin for 8 hours (Figure 2B); suggesting to choose 100 nM insulin for further experiments. A two fold increase in PKM2 mRNA and protein expression was observed upon 100 nM insulin treatment for 8 hours (Figure 2C-E), whereas, PKL expression remained unaltered under similar conditions (Figure 2D). Insulin treatment up-regulated PKM2 in H1299 (lung cancer) and PC3 (prostate cancer) cells also (Additional file 2: Figure S2), replicating the observations made in HepG2 cells.

PI3K/mTOR signalling was inhibited to find out if insulin-induced PKM2 up-regulation is PI3K dependent. PI3K is the chief mediator of insulin signalling in liver and is frequently mutated in liver and other cancers [26-28]. As a result of PI3K/mTOR inhibition, PKM2 expression was down-regulated (Figure 2C-E). These results confirmed that insulin regulates PKM2 expression through PI3K/mTOR pathway. To elucidate how PI3K/mTOR promoted PKM2 expression, the role of HIF1α protein, a transcription factor that binds to hypoxia response element (HRE) in PKM2 gene to induce its expression, was examined [29,30]. Insulin induced HIF1α expression in a PI3K/mTOR sensitive manner, as was evident from the decrease in HIF1α protein after treatment with PI3K/mTOR inhibitors (Figure 2D). Further, to corroborate this observation, HIF1α was silenced as well as induced, using siRNA and cobalt chloride (CoCl_2_) treatment, respectively, to observe the effect on PKM2 expression [31,32]. Expectedly, HIF1α silencing led to a decreased PKM2 expression; whereas CoCl_2_-induced HIF1α accumulation increased PKM2 expression (Figure 2F), confirming that HIF1α is crucial for PKM2 up-regulation. These results demonstrated that insulin upregulates PKM2 expression through PI3K/mTOR dependent induction of HIF1α transcription factor.

### Insulin inhibits PKM2 activity, in a PI3K/mTOR independent manner, by promoting subunit dissociation

**Figure 3:**
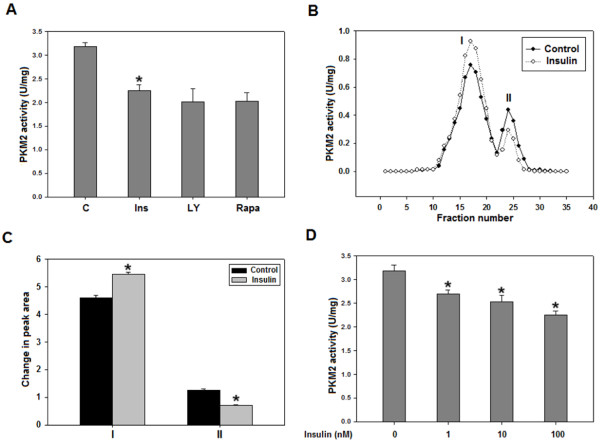
**PKM2 activity decreased as a result of subunit dissociation upon insulin treatment, in a PI3K/mTOR independent manner. (A)** 100 nM insulin treatment of 15 minutes reduced PKM2 activity in HepG2 cells by ~30%. Pretreatment with PI3K/mTOR inhibitors (50 μM LY294002 or 20 nM rapamycin for 30 minutes) did not significantly alter PKM2 activity. For activity measurement: NADH/LDH coupled assay was used to monitor decrease in OD due to oxidation of NADH at 340 nm. **(B)** Glycerol density gradient centrifugation was used to separate oligomeric forms of PKM2. Activity was assessed in different fractions to detect any shift in peaks i.e. subunit dissociation. Rise in peak I with concomitant fall in peak II is suggestive of disruption of highly oligomerised PKM2 (tetramer) in less oligomerised forms. Activity in a fraction corresponds to the respective amount of particular oligomeric form. **(C)** Relative peak areas show significant shift in peaks I and II. As tetrameric PKM2 has optimal activity [15], it can be concluded that PKM2 activity decreased as a result of dissociation of active tetramers. **(D)** Dose-dependent decrease in PKM2 activity upon insulin treatment. Data is expressed as mean ± SE. *P ≤ 0.05*.*

PKM2 activity is crucial in determining the glycolytic flux; it was pertinent therefore to examine the potential role of insulin in altering PKM2 activity. Interestingly, ~30% drop in PKM2 activity was observed in HepG2 cells within 15 minutes after insulin treatment with no further decrease on increasing the duration of treatment (Figure 3A, Additional file 3: Figure S3). However, PI3K/mTOR inhibition did not reverse insulin-induced decrease in PKM2 activity (Figure 3A). Insulin-treated H1299 and PC3 cells also exhibited decrease in PKM2 activity (Additional file 4: Figure S4). To analyse if the insulin-induced drop in PKM2 activity is due to subunit dissociation, which governs the overall activity of PKM2 [9,11,15,33,34], glycerol density gradient centrifugation was performed using HepG2 cell lysate. Density gradient centrifugation is known to separate different oligomeric forms of an enzyme with highest quaternary form at the bottom of the gradient and the magnitude of activity representing the respective content of an oligomeric form [35]. PKM2 activity was measured in fractions collected from top to bottom of the glycerol gradient. Observation of two peaks suggested two oligomeric forms of PKM2 and the vertical shift in peaks indicated subunit dissociation (Figure 3B). As evident from relative peak areas, a significant fall in peak II with concomitant increase in peak I, compared to control peaks, was observed upon insulin treatment (Figure 3C). Dose dependent insulin treatment showed that even 1nM insulin treatment decreased PKM2 activity significantly with maximum decrease on 100 nM insulin (Figure 3D). Results, thus, suggested that decrease in PKM2 activity was due to subunit dissociation upon insulin treatment. As expected, PI3K/mTOR inhibition did not abolish the effect of insulin on PKM2 oligomeric status (Additional file 5: Figure S5). In an attempt to understand how insulin promoted subunit dissociation, we checked the tyrosine 105 phosphorylation of PKM2, which is known to diminish its activity by disrupting the formation of active tetramer [33], but, no change in phosphorylation was observed following insulin treatment (Additional file 6: Figure S6 and Additional file 7: Figure S7). These results suggested the role of some other insulin-induced factors in PKM2 activity regulation.

### Evidence for the involvement of ROS in inhibition of PKM2 activity

**Figure 4:**
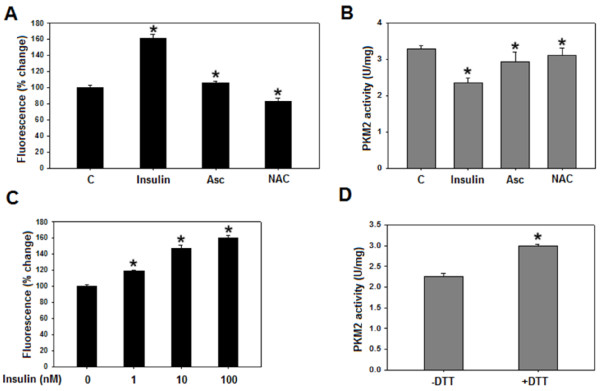
**Ascorbate or NAC pretreatment decreases insulin induced ROS and increases PKM2 activity. (A)** Serum starved HepG2 cells, pretreated with 200 μM ascorbate or 5 mM NAC for 1 hour [41], were incubated with DCFH-DA for 30 minutes at 37°C (in dark) followed by 100 nM insulin treatment for 15 minutes. DCFH-DA fluorescence was measured to assess production of ROS (see Materials and methods). Insulin increased ROS production by ~60% as compared to serum starved control. However, ascorbate or NAC pretreatment significantly decreased insulin induced ROS. **(B)** PKM2 activity increased in ascorbate or NAC pretreated cells compared to untreated control. **(C)** Dose dependent increase in ROS production by insulin. **(D)** PKM2 activity from insulin treated cells in absence and presence of 1mM DTT. Reversal of insulin-induced decrease in PKM2 activity by DTT suggests the possibility of oxidation of PKM2 by ROS. Data is expressed as mean ± SE. *P ≤ 0.05*.*

To find out the factor responsible for inhibition of PKM2 activity in HepG2 cells, the possible role of insulin-induced ROS was analysed. ROS is known to regulate the activity of several proteins through oxidation, we thus investigated if insulin-induced-ROS affected PKM2 activity through its possible oxidation. Insulin treatment increased the *in-vitro* production of ROS by ~60%, as assessed by fluorescence spectrophotometry (Figure 4A). Pretreatment with ascorbate (Asc) or N-acetyl-L-cysteine (NAC), well-known ROS scavengers [36-41], reduced insulin-induced-ROS markedly by ~50-70%. This observation was replicated in H1299 and PC3 cells (Additional file 8: Figure S8). Interestingly, ascorbate or NAC pretreatment, followed by incubation with insulin, reversed the insulin-induced decrease in PKM2 activity in HepG2 (Figure 4B), H1299 and PC3 (Additional file 4: Figure S4) cells, suggesting the role of insulin-induced-ROS in PKM2 activity inhibition. Further, dose-dependent increase in ROS production by insulin correlated with dose-dependent decrease in PKM2 activity (compare Figures 3D and 4C). To understand how ROS contributed to reduction in PKM2 activity, the possibility of ROS-induced- oxidation of PKM2 was studied. Addition of dithiothreitol (DTT), a strong reducing agent, to activity reaction mixture abolished insulin-induced-decrease in PKM2 activity (Figure 4D). In fact, addition of DTT increased PKM2 activity to the levels similar to that of control without insulin treatment (Figure 4D and B). These data indicated the role of ROS-induced-oxidation of PKM2 [34], resulting in its activity-inhibition.

### PKM2 up-regulation is crucial in insulin-induced aerobic glycolysis

**Figure 5:**
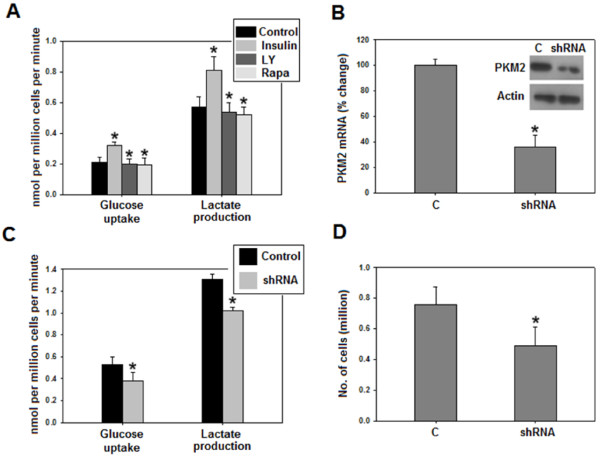
**Insulin promoted aerobic glycolysis, partly, by upregulating PKM2.** Media used by insulin treated (100 nM for 8 hours) or untreated cells was collected for measurement of glucose uptake and lactate production as described in materials and methods section. **(A)** Insulin promoted glucose uptake and lactate production in a PI3K/mTOR sensitive manner. **(B)** PKM2 knockdown efficiency as checked by real time and Western blotting. **(C)** Glucose uptake and lactate production decreased on silencing PKM2 in PTEN negative PC3 cells; indicating that insulin stimulated aerobic glycolysis, partly, through PKM2 up-regulation. **(D)** Cellular proliferation decreased on PKM2 knockdown. *C-serum starved cells*. Data is expressed as mean ± SE. *P ≤ 0.05.

PKM2 expression has been suggested to promote aerobic glycolysis [12,30], which is characterized by high glucose uptake and lactate production even in presence of oxygen, and is believed to be the hallmark of nearly all cancer cells [4]. Both glucose uptake and production of lactate were substantially enhanced in cells treated with insulin as compared to untreated control (Figure 5A). Further, inhibition of PI3K/mTOR pathway reduced aerobic glycolysis which is consistent with earlier reports (Figure 5A) [42,43]. To show that PI3K/mTOR dependent PKM2 up-regulation contributed to insulin-induced augmentation in aerobic glycolysis, PKM2 was knocked down in cells with hyperactive PI3K/mTOR signalling (to mimic insulin-induced activation of PI3K/mTOR), followed by an assessment of glucose uptake and lactate production, approximately 48 hours after transfecting control shRNA or PKM2-shRNA (to knock down PKM2 expression). Interestingly, silencing of PKM2 partially inhibited glucose uptake and lactate production, signifying that PKM2 is required for aerobic glycolysis (Figure 5B-C). Moreover, PKM2 knockdown also retarded cellular proliferation (Figure 5D), consistent with our previous observation [44].

### Glycolytic intermediates and NADPH accumulated as a result of decreased PKM2 activity

**Figure 6:**
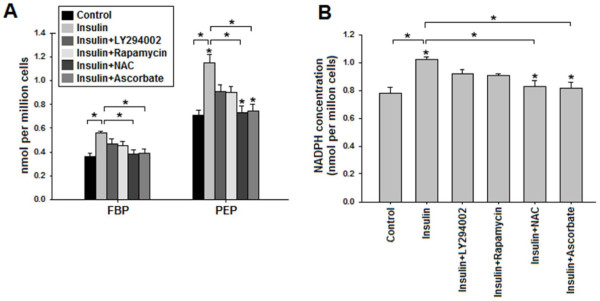
**Increased glycolytic pooling and NADPH accumulation due to insulin-induced PKM2 activity inhibition.** Serum starved HepG2 cells were treated with or without 100 nM insulin for 15 minutes or pretreated with 50 μM LY294002 or 20 nM rapamycin or 5 mM NAC or 200 μM for 30 minutes followed by 100 nM insulin treatment. Intracellular PEP, FBP and NADPH were extracted and measured as described in materials and methods section. **(A)** Increased accumulation of PEP (~1.7 fold) and FBP (~1.6 fold) was observed in insulin stimulated cells. LY294002 and rapamycin decreased glycolytic pooling modestly, however ROS scavengers NAC and ascorbate substantially decreased glycolytic pooling of FBP and PEP. **(B)** NADPH accumulated in insulin treated cells, suggesting enhanced anabolic synthesis via PPP [8,9]. Pretreatment with PI3K/mTOR inhibitors decreased NADPH accumulation; however, pretreatment with ROS scavengers reduced NADPH accumulation to greater extent, bringing NADPH levels similar to that of control. These results suggest that insulin promoted accumulation of glycolytic intermediates mainly by decreasing PKM2 activity, and also indicates the diversion of glucose flux towards PPP [8,9]. Actual change in intracellular concentrations is expressed as nmol per million cells. Data is expressed as mean ± SE. *P ≤ 0.05.

Characteristic accumulation of glycolytic intermediates, as a result of decreased PKM2 activity, is another key feature of cancer cells and has been shown to be important for anabolic synthesis required for tumor growth [8,11]. To explore the implications of insulin-induced suppression of PKM2 activity on glycolytic pooling, intracellular levels of fructose-1,6-bisphosphate (FBP) and phosphoenolpyruvate (PEP), the glycolytic metabolites upstream of PKM2, were measured [15]. Consequently, an increased build-up of both metabolites was observed compared to control (Figure 6A). Glycolytic pooling is known to facilitate pentose phosphate pathway for macromolecular synthesis [8,9,13]. Therefore, intracellular levels of NADPH, which is produced as a result of PPP and provides reducing power for macromolecular synthesis [45], were determined. Accumulation of NADPH increased significantly (*P* < 0.05) in insulin treated cells (Figure 6B).

To demonstrate that decreased PKM2 activity is contributing to the observed accumulation of FBP, PEP and NADPH, PI3K/mTOR pathway was inhibited as it increases glucose uptake which might have contributed to observed accumulation. PI3K/mTOR inhibition did not completely block the insulin-induced pooling of FBP, PEP and NADPH. However, pretreatment with ROS scavengers NAC or ascorbate abolished the insulin-induced glycolytic pooling and accumulation of NADPH. These results indicated that insulin promoted accumulation of glycolytic intermediates and NADPH through decreased PKM2 activity.

## Discussion

In recent years, PKM2 has emerged as a key regulator of cancer metabolism. Considering the PKM2-mediated pro-cancerous effects of insulin, our results seemingly provide a mechanistic understanding into insulin’s role in cancer metabolism.

The predominance of PKM2 over other PK isoforms in HepG2, H1299 and PC3 cells (Figure 1 and Additional file 1: Figure S1) [30,46], indicated the importance of this PK isoform in cancer, and is consistent with the notion that switch to PKM2 isoform is required for cancer progression [12]. The insulin induced-up- regulation of PKM2, with evident increase at as low as 1 nM insulin (Figure 2), is an important observation, since PKM2 has been reported essential for aerobic glycolysis and tumor growth [30]. PI3K-dependence of PKM2 expression supports the notion that PI3K pathway is central to insulin signalling in cancer cells and correlates with high frequency of PI3K mutations in several cancer types, which may lead to increased expression of PKM2 [26,27]. PI3K/mTOR dependent induction of HIF1α on insulin treatment, and CoCl_2_-induced concomitant HIF1 α and PKM2 accumulation, along with HIF1α silencing results (with decreased PKM2 expression) clearly suggested that insulin promoted PKM2 expression through HIF1α (Figure 2D and F). Our results are consistent with previous observations of insulin up-regulation of PKM2 in adipocytes [47] and decreased PKM2 expression on PI3K/mTOR inhibition [44,48]. These results also explain the recent observation of high PKM2 in PTEN (negative regulator of PI3K pathway) null fatty liver cells [49]. Decreased aerobic glycolysis on PKM2 knock-down in PTEN deficient (hyper-activated PI3K/mTOR signalling) PC3 cells suggested that insulin promoted aerobic glycolysis, at least in part, through PKM2 (Figure 5). Importance of PKM2 in aerobic glycolysis could be realized from the observation that PKM2 transactivates expression of genes like glucose transporter-1 (GLUT1) and lactate dehydrogenase-A (LDHA), required for glucose uptake and lactate production respectively [29]. Apparently, these results present an important mechanism which may underlie insulin’s role in carcinogenesis.

Decreased PKM2 activity fuels macromolecular synthesis by accumulating glycolytic intermediates that are precursors for PPP. Suppression of PKM2 activity presents yet another dimension of insulin’s role in promotion of cancer metabolism. Rise in peak I with simultaneous fall in peak II suggested subunit dissociation leading to increased formation of low activity oligomeric form of PKM2 (Peak I in Figure 3B), thus justifying the observed decrease in PKM2 activity (Figure 3A). Intriguingly, activity of PKM2 was not affected on PI3K/mTOR inhibition (Figure 3A and Additional file 6: Figure S5), indicating the involvement of other factors in activity regulation. Increased PKM2 activity in ascorbate or NAC pretreated cells suggests the involvement of ROS in activity regulation (Figure 4 and Additional file 4: Figure S4). Reversal of insulin-induced decrease in PKM2 activity by DTT suggests the possibility of ROS-induced oxidation of cysteine residues in PKM2 [34]. Correlation between dose dependent changes in PKM2 activity and ROS production further support our conclusion of ROS mediated PKM2 activity inhibition (Figures 3D and 4C). Notably, ROS induced decrease in PKM2 activity has been linked with ability of lung cancer cells to withstand oxidative stress [34]. However, further research is needed to support this observation in liver and prostate cancer cells.

The decrease in the activity of PKM2, positioned at the end of glycolytic sequence, in cancer cells is suggested to accumulate important glycolytic intermediates required for cellular growth. On the contrary, a decrease in the activity of enzyme, like hexokinase, which appear earlier in glycolytic sequence, does not provide the same advantage to the cancer cells; thus the biosynthetic processes required for cellular growth would be inhibited. Accumulation of glycolytic intermediates PEP and FBP (Figure 6) indicated the diversion of glucose flux towards biosynthetic pathway PPP [8], an observation which was confirmed when PPP product NADPH accumulated upon insulin treatment (Figure 6B). The decrease in glycolytic pooling and NADPH accumulation on PI3K/mTOR inhibition correlated with glucose uptake regulation by PI3K/mTOR pathway (Figures 5A and 6). Interestingly, metabolite accumulation decreased almost to the levels of untreated control upon treatment with ROS scavengers NAC or ascorbate, suggesting a crucial role of decreased PKM2 activity in insulin-induced metabolite build-up. ROS scavenging increased PKM2 activity which negatively affected metabolite pooling and macromolecular synthesis (Figures 4B and 6).

**Figure 7:**
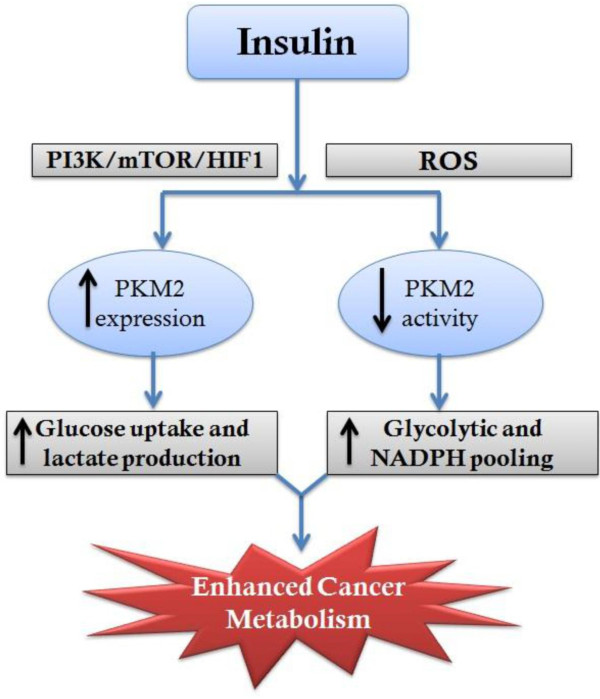
**Graphical summary.** Effect of insulin on PKM2 expression (through PI3K/mTOR/HIF axis) and activity (ROS-dependent), resulting in amplified cancer metabolism.

On insulin treatment a maximum PKM2 expression was observed at 8 hours; whereas a decrease in activity was observed within 15 minutes, which was almost constant till 8 hours. Since no further decrease in activity was observed by prolonging insulin treatment till 8 hours (Additional file 3: Figure S3), we chose 15 minutes as the earliest time point at which PKM2 activity decreased. The up-regulation of PKM2 expression with concomitant decrease in activity was associated with enhanced aerobic glycolysis and increased macromolecular synthesis (Figures 4 and 5). PKM2 expression is critical for aerobic glycolysis as it transactivates expression of GLUT1 (glucose transporter) and LDHA (lactate dehydrogenase) [29]. We have shown that knockdown of PKM2 expression decreased aerobic glycolysis (Figure 5C) while inhibition of PKM2 activity promoted pooling of glycolytic intermediates which are then shunted to PPP for anabolic synthesis [8,13,33,34]. Apparently, dual regulation of PKM2 expression and activity by insulin ensures the promotion of both aerobic glycolysis and anabolic synthesis, required for cancer cell proliferation (Figure 7). This possibly explains the link between high insulin levels and elevated cancer risk. Moreover, reduced PKM2 activity might explain liver cirrhosis due to fatty liver since PKM2 activity reduction is known to promote lipid synthesis [13].

## Conclusion

Our study highlights previously unknown PKM2-mediated effects of insulin in promotion of cancer metabolism, which probably explains the observations of increased cancer risk under hyperinsulinemic condition [17,24,50].

## Materials and methods

### Cell culture, drug treatment, knockdown and proliferation studies

HepG2 and PC3 cell lines were procured from the National Centre for Cell Science, Pune, India. H1299 cells were a kind gift from Dr. Uttam Pati, School of Biotechnology, Jawaharlal Nehru University. All the cell lines were maintained in DMEM (Sigma) with 10% heat-inactivated FBS (Biowest, France), 1% penicillin/streptomycin (Sigma) at 37°C and 5% CO_2_ in a humified atmosphere (Heraeus, UK). Cells were grown in monolayer and passaged routinely two-three times a week. For drug treatment; LY294002 (Sigma) and rapamycin (Sigma) were dissolved in DMSO; single use aliquots were stored at −80°C For insulin treatment: cells were seeded in triplicate at a density of 0.4 million cells/well of six well plates, serum starved for 20–24 hours and then stimulated with insulin (Sigma) in presence or absence of inhibitors. DMSO treated cells were used as mock control. For PKM2 knockdown: short hairpin RNA (shRNA) constructs in lentiviral pGIPZ vector were purchased from Open Biosystems. Control and shRNA containing pGIPZ vectors were transfected using polymer based transfection reagent- Arrestin from Thermo-Scientific, as per manufacturer’s instructions. Knock down efficiency was then checked by real time PCR and Western blotting. For HIF1α silencing: pre-designed siRNA was used and transfected using Lipofectamine (Invitrogen) as per manufacturer’s protocol and efficiency was checked by Western blotting. For proliferation assay: cells were counted before and after 48 hours of transfection, using a hemocytometer.

### RNA isolation, cDNA preparation and real time PCR

Cellular RNA was extracted from cell lines using TRIzol (Sigma), according to manufacturer’s protocol. RNA quality was analyzed by A_260_/A_280_ absorbance and by electrophoresis on a 1.2% agarose formaldehyde gel. 3–4 μg of total RNA was reverse transcribed into single stranded DNA using cDNA preparation kit (Applied Biosystems, USA). Commercially available Taqman gene expression assay (Applied Biosystems, USA) was used for quantitating mRNA levels of PKM2. β-actin was used as endogenous control. Real time PCR was carried out on ABI Prism 7000 Sequence Detection System (Applied Biosystems). ΔΔC_t_ (Cycle threshold) method of relative quantification was used to calculate fold change in gene expression by SDS 1.1 RQ software (Applied Biosystems).

### Cell lysate preparation, protein estimation and Western blotting

Whole cell lysate was prepared by incubating cells, on ice for 30 minutes, in buffer containing 50 mM Tris pH 7.2, 150 mM NaCl, 0.5% sodium deoxycholate, 10% glycerol, 1% Triton X-100, 0.1% SDS, 1 mM DTT, 1 mM PMSF, 5 mM NaF, 1 mM NaV, phosphatase inhibitor cocktail (Sigma), 4 μg/ml aprotinin, 4 μg/ml leupeptin and 4 μg/ml pepstatin (Sigma). The lysate was centrifuged at high speed in a cooling centrifuge (CM 12, Remi, India) for 30 minutes and supernatant was collected in pre-chilled fresh tubes. Protein concentration was estimated using BCA method as per manufacturer protocol (Thermo Scientific). Proteins were separated on 8% SDS-PAGE, transferred to nitrocellulose membrane (mdi) at 4°C (wet transfer) and probed with primary antibodies. Membrane was incubated with appropriate secondary antibody for one hour at room temperature and proteins were detected using Luminata forte (Millipore). Primary antibodies used were: anti-HIF1α (Novus Biologicals, USA), anti PKM2, anti-phospho-PKM2 (Tyr105), anti-AKT, anti-phospho-AKT, anti-phosphoS6, anti-S6 protein and anti-β-actin (Cell Signalling Technology).

### PKM2 activity assay and glycerol gradient centrifugation

For activity, cells were lysed in buffer as described previously [12]. Activity was measured using NADH/lactate dehydrogenase (LDH) coupled assay. Decrease in OD at 340 nm due to oxidation of NADH was monitored using a double beam spectrophotometer (UV-1800, Shimadzu). Reaction was started by adding 2 μg cell lysate to mixture containing 50 mM Tris pH 7.5, 100 mM KCl, 5 mM MgCl_2_, 1.25 mM ADP, 0.5 mM PEP, 0.28 mM NADH and 8 units of LDH. Specific activity per mg of cell lysate was calculated as:

$$U/\mathrm{mg}=\frac{OD_{340}/\min}{6.22\times \mathrm{mg}\;\mathrm{lysate}/\mathrm{ml}\;\mathrm{reaction}\;\mathrm{mixture}}$$

U/mg = specific enzyme activity per mg of protein.

OD_340_ = Change in absorbance due to oxidation of NADH in one minute at 340 nm wavelength.

For glycerol gradient experiment, 500 μg of cell lysate protein was loaded on the top of 11-25% glycerol gradient and centrifuged at 45000 rpm for 16 hours at 4°C in SW55Ti rotor (Beckman Coulter) and rest of the procedure was followed as described [16].

### Metabolites, glucose and lactate measurement

Metabolite extract was prepared from 20 million cells in 0.5 ml of chilled 90% ethanol containing 0.2% formic acid and centrifuged at 15000 rpm in a refrigerated centrifuge. Supernatant was dried using nitrogen flow and then reconstituted in 0.2 ml of MilliQ water. PEP was assessed using NADH/LDH coupled assay as mentioned above with 30 ng of recombinant PKM2. FBP was measured as described [51]. For both FBP and PEP, concentration was determined against standard curve. NADPH was analyzed using kit from BioVision- USA, as per the manufacturer’s protocol. For glucose and lactate: media was collected from wells; spun down at high speed to remove any cell debris, deproteinized using TCA, pH was adjusted between 7.0-7.5 and then glucose uptake was analyzed using glucose assay kit (Sigma), according to manufacturer’s protocol. Lactate and was analysed using kit (BioVision, USA) as per manufacturer’s instructions. All the measurements were normalized to cell numbers.

### Ascorbate, NAC treatment and ROS analysis

Cells pretreated with or without ascorbate or NAC (Sigma) were incubated with DCFH-DA for 30 minutes in dark at 37°C, followed by insulin stimulation. Cells were then washed with PBS, trypsinized and resuspended in 0.5 ml PBS, kept on ice, in dark until ROS analysis by fluorescence spectrophotometry. Excitation and emission wavelengths used were 500 nm and 510 nm respectively.

### Statistical analysis

Each experiment was performed in triplicate. All experiments were repeated at least 3 times. Significance was calculated using *student’s t-test*. *P* value less than 0.05 was considered statistically significant.

## Abbreviations

PKM2: Pyruvate kinase M2; PI3K: Phosphoinositide-3-kinase; mTOR: Mammalian target of rapamycin; PEP: Phosphoenolpyruvate; FBP: Fructose 1,6-bisphosphate; PPP: Pentose phosphate pathway; NADPH: Nicotinamide adenine dinucleotide phosphate; ROS: Reactive oxygen species; NAC: N-acetyl-L-cysteine

## Competing interests

The authors declare that they have no competing interests.

## Authors’ contributions

MAI designed study; acquired, analysed and interpreted data; performed statistical analysis and drafted manuscript. FAS participated in acquisition of data, statistical analysis and manuscript preparation. VG, SC, PG, BK, SM and NC participated in experimental data acquisition and revision of manuscript. RNKB conceived the study, critically reviewed manuscript for intellectual content and gave final approval for submission. All authors read and approved the final version of manuscript.

## Supplementary Material

Additional file 1: Figure S1 PKM2 is the predominant isoform in H1299 and PC3 cell. PKM1 expression is negligibly low. Click here for file

Additional file 2: Figure S2 Insulin up-regulated PKM2 expression in H1299 and PC3 cells *(C = Control, I = 100 nM insulin).* Click here for file

Additional file 3: Figure S3 PKM2 activity after different time points of 100 nM insulin treatment. Data is expressed as mean ± SE. Click here for file

Additional file 4: Figure S4 Insulin treatement decreased PKM2 activity in H1299 and PC3 cells. Pretreatment with ROS scavenger- NAC, reversed insulin-induced decrease in activity. Data is expressed as mean ± SE. *P ≤ 0.05. Click here for file

Additional file 5: Figure S5 Glycerol gradient of cells pre-treated with DMSO or 50 μM LY294002 or 20 nM rapamycin, followed by 100 nM insulin treatment for 15 minutes. Click here for file

Additional file 6: Figure S6 Representative Western blot showing no change in phosphor-tyr-105-PKM2 upon insulin treatment (100 nM for 15 minutes) and inhibition with 50 μM LY294002 and 20 nM rapamycin, in HepG2 cells. Click here for file

Additional file 7: Figure S7 Immunoblot showing no change in phosphor-tyr-105-PKM2 upon insulin treatment (100 nM for 15 minutes) in H1299 and PC3 cells. Click here for file

Additional file 8: Figure S8 Insulin treatement increase ROS in H1299 and PC3 cells. Pretreatment with 5 mM NAC decreased insulin-induced ROS. Data is expressed as mean ± SE. *P ≤ 0.05. Click here for file
