# Supplementary figures and images for: Insulin enhances metabolic capacities of cancer cells by dual regulation of glycolytic enzyme pyruvate kinase M2
Source: Mol Cancer. 2013 Jul 9;12:72. doi: 10.1186/1476-4598-12-72 (PMC3710280; doi:10.1186/1476-4598-12-72)

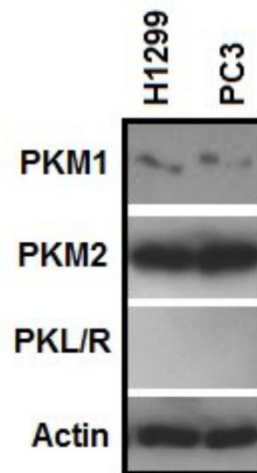

**Fig S1.** PKM2 is the predominant isoform in H1299 and PC3 cell. PKM1 expression is negligibly low.

Supplement: Additional file 1: Figure S1 — PKM2 is the predominant isoform in H1299 and PC3 cell. PKM1 expression is negligibly low. [file 1476-4598-12-72-S1.pdf]

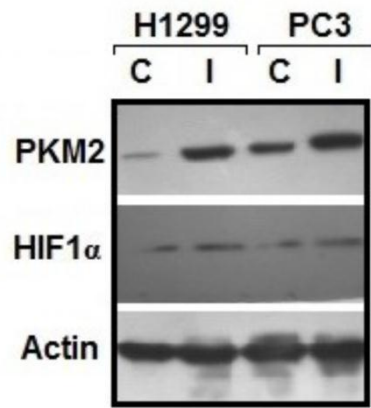

**Fig S2.** Insulin up-regulated PKM2 expression in H1299 and PC3 cells. (*C*= Control, *I*= 100 nM insulin).

Supplement: Additional file 2: Figure S2 — Insulin up-regulated PKM2 expression in H1299 and PC3 cells (C = Control, I = 100 nM insulin). [file 1476-4598-12-72-S2.pdf]
