# Supplementary material for: Insulin enhances metabolic capacities of cancer cells by dual regulation of glycolytic enzyme pyruvate kinase M2
Source: Mol Cancer. 2013 Jul 9;12:72. doi: 10.1186/1476-4598-12-72 (PMC3710280; doi:10.1186/1476-4598-12-72)
Supplement: Additional file 3: Figure S3 — PKM2 activity after different time points of 100 nM insulin treatment. Data is expressed as mean ± SE. [file 1476-4598-12-72-S3.pdf]

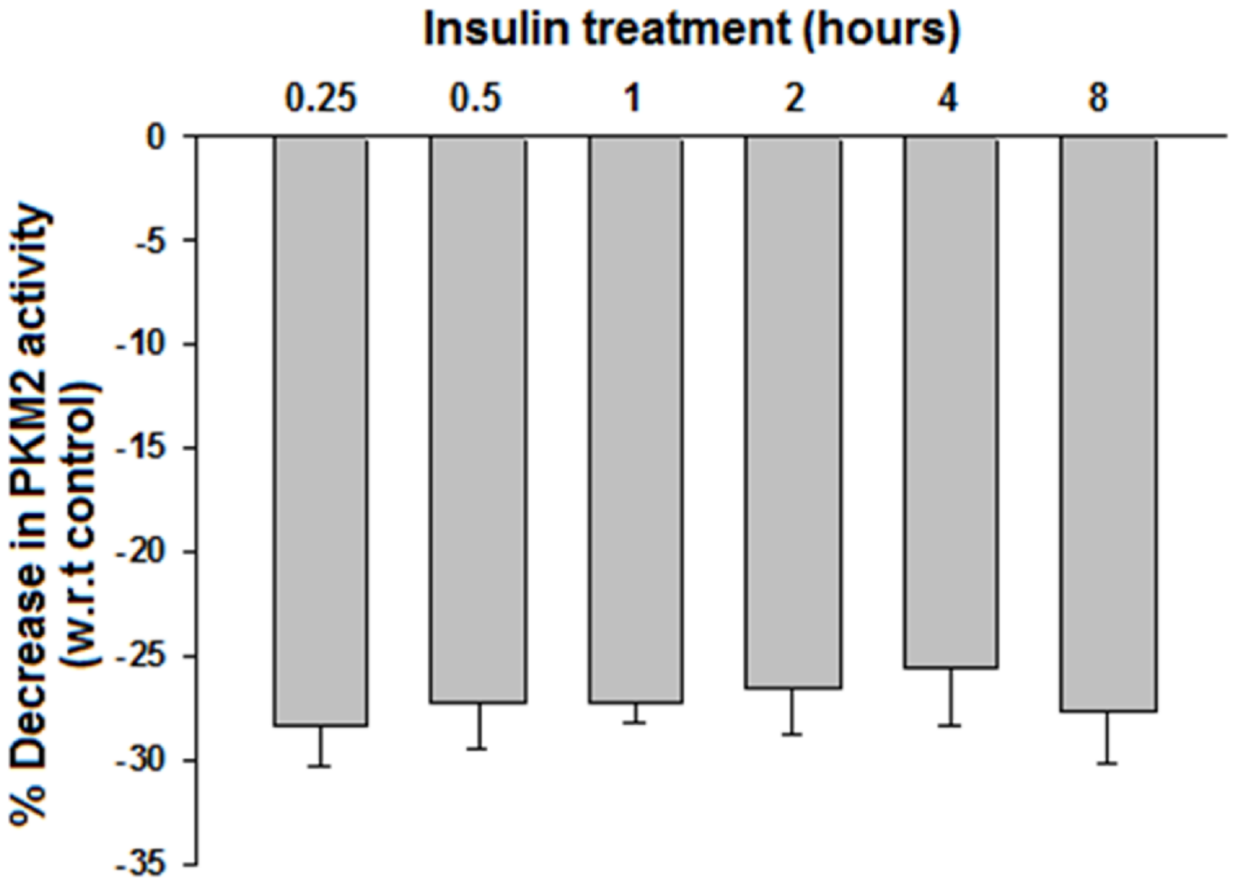

Fig. S3. PKM2 activity after different time points of 100 nM insulin treatment. Data is expressed as mean ±SE.
