# Supplementary material for: Insulin enhances metabolic capacities of cancer cells by dual regulation of glycolytic enzyme pyruvate kinase M2
Source: Mol Cancer. 2013 Jul 9;12:72. doi: 10.1186/1476-4598-12-72 (PMC3710280; doi:10.1186/1476-4598-12-72)
Supplement: Additional file 4: Figure S4 — Insulin treatement decreased PKM2 activity in H1299 and PC3 cells. Pretreatment with ROS scavenger- NAC, reversed insulin-induced decrease in activity. Data is expressed as mean ± SE. *P ≤ 0.05. [file 1476-4598-12-72-S4.pdf]

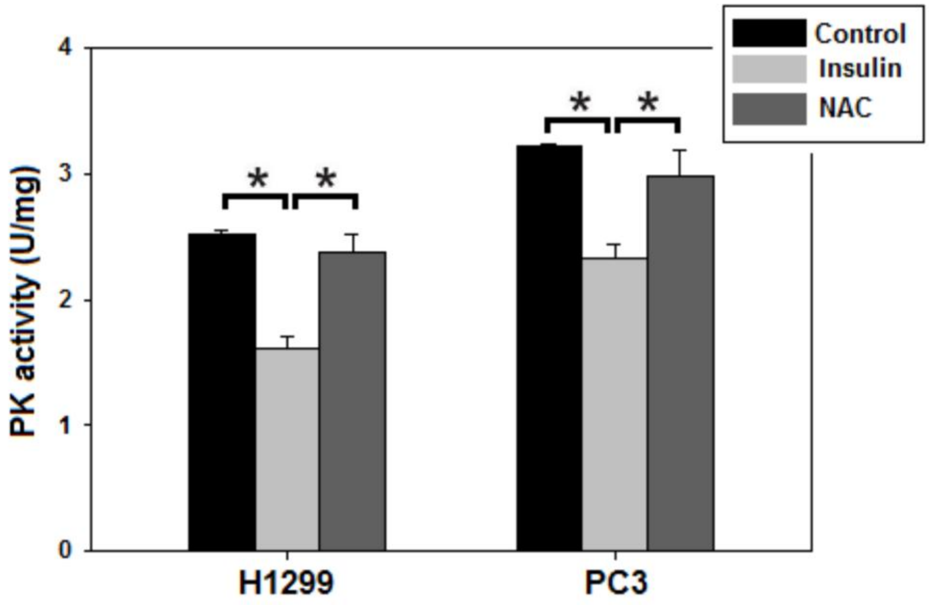

Fig. S4. Insulin treatement decreased PKM2 activity in H1299 and PC3 cells. Pretreatment with ROS scavenger- NAC, reversed insulin-induced decrease in activity. Data is expressed as mean ±SE. *P≤ 0.05.
