# Supplementary material for: Insulin enhances metabolic capacities of cancer cells by dual regulation of glycolytic enzyme pyruvate kinase M2
Source: Mol Cancer. 2013 Jul 9;12:72. doi: 10.1186/1476-4598-12-72 (PMC3710280; doi:10.1186/1476-4598-12-72)
Supplement: Additional file 5: Figure S5 — Glycerol gradient of cells pre-treated with DMSO or 50 μM LY294002 or 20 nM rapamycin, followed by 100 nM insulin treatment for 15 minutes. [file 1476-4598-12-72-S5.pdf]

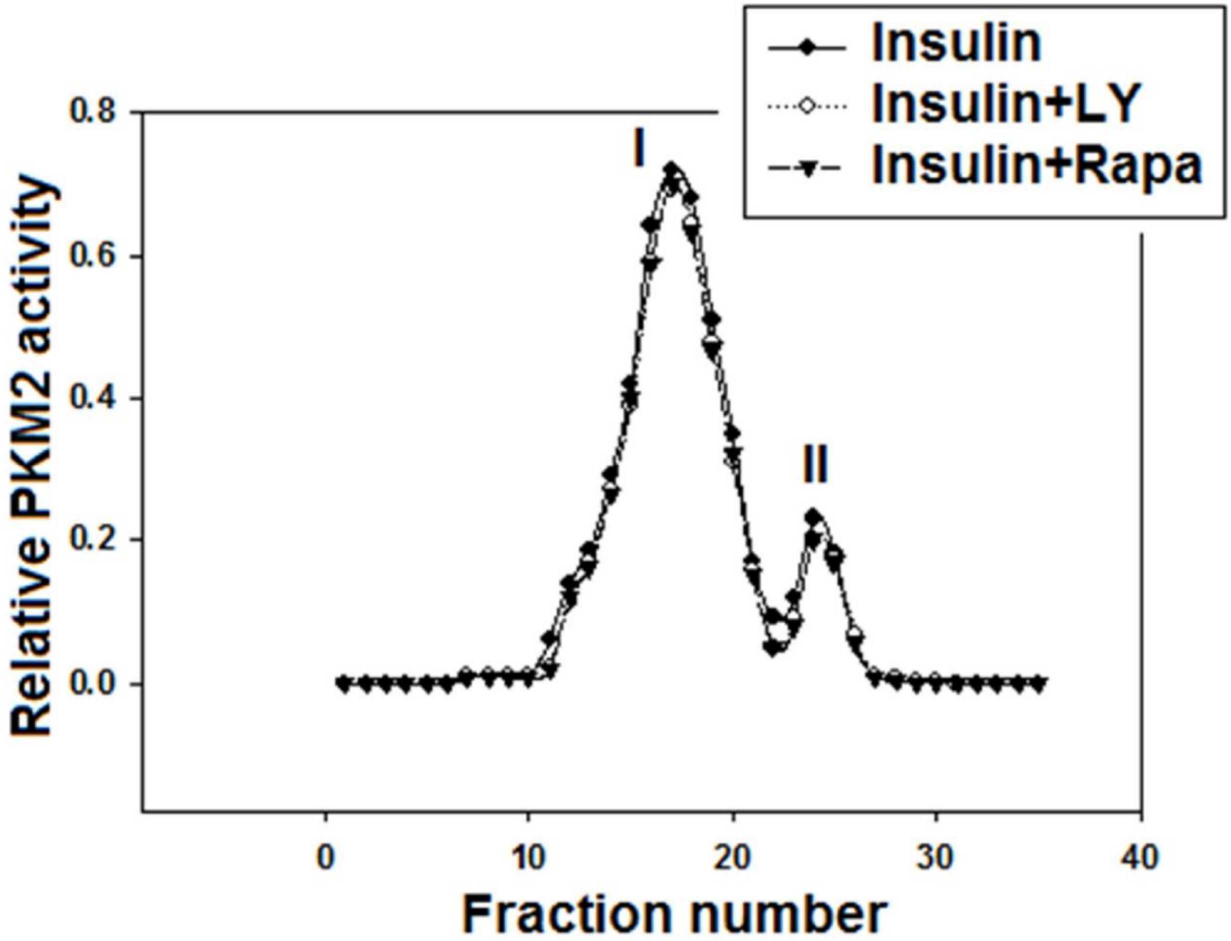

Fig. S5 Glycerol gradient of cells pre-treated with DMSO or 50 µM LY294002 or 20 nM rapamycin, followed by 100 nM insulin treatment for 15 minutes.
