# Supplementary material for: Insulin enhances metabolic capacities of cancer cells by dual regulation of glycolytic enzyme pyruvate kinase M2
Source: Mol Cancer. 2013 Jul 9;12:72. doi: 10.1186/1476-4598-12-72 (PMC3710280; doi:10.1186/1476-4598-12-72)
Supplement: Additional file 7: Figure S7 — Immunoblot showing no change in phosphor-tyr-105-PKM2 upon insulin treatment (100 nM for 15 minutes) in H1299 and PC3 cells. [file 1476-4598-12-72-S7.pdf]

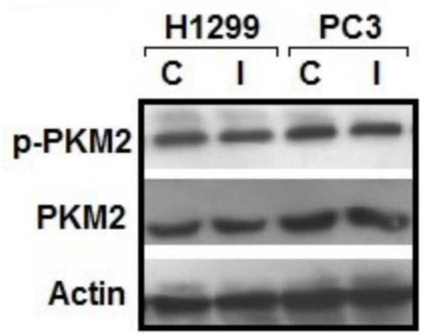

Fig S7. Immunoblot showing no change in phosphor-tyr-105-PKM2 upon insulin treatment (100 nM for 15 minutes) in H1299 and PC3 cells.
